# Species Specific Behavioural Patterns (Digging and Swimming) and Reaction to Novel Objects in Wild Type, Wistar, Sprague-Dawley and Brown Norway Rats

**DOI:** 10.1371/journal.pone.0040642

**Published:** 2012-07-16

**Authors:** Rafał Stryjek, Klaudia Modlińska, Wojciech Pisula

**Affiliations:** 1 Institute of Psychology, Polish Academy of Sciences, Warsaw, Poland; 2 Helena Chodkowska University of Management and Law, Warsaw, Poland; Oxford Brookes University, United Kingdom

## Abstract

**Background:**

The purpose of the present study was to analyse species-specific forms of behaviour (digging and swimming) and response to novelty in laboratory rats and their wild type counterparts at a very early stage of laboratorization. Three behavioural phenomena were taken into account: burrowing, spontaneous swimming, and neophobic behaviour.

**Principal Findings:**

Wild-type rats and three strains of laboratory rats were involved in experiments: Warsaw-Wild-Captive-Pisula-Stryjek (WWCPS), Wistar, Sprague-Dawley, and Brown Norway rats were compared in spontaneous swimming test, while WWCPS and Wistar rats were studied in burrowing and neophobia experiments. Wild rats were found to be faster at building tunnels than Wistar rats and at constructing more complex burrow systems. The experiment on neophobia showed that Wistar rats exhibited less neophobic responses and were more often trapped. WWCPS rats showed highly neophobic behaviour and were rarely trapped in this experiment. The experiment on swimming showed that WWCPS rats showed more complex water tank related activity than their laboratory counterparts. They swam and explored under surface environment.

**Conclusions:**

The three experiments showed profound behavioural differences in quasi-natural forms of behaviour between wild type rats (WWCPS) and three laboratory strains frequently used in behavioural studies.

## Introduction

So far, significant morphological and behavioural differences between laboratory and wild rats have been reported in a number of important domains. Lockard [1] suggested that the two groups are anatomically different. Laboratory rats are larger [2], more bulky, have a weaker bone structure, smaller internal organs such as the brain, heart, liver, spleen, etc., and a poorer sense of smell [3]. Multiple differences are also apparent in terms of behaviour. Wild rats demonstrate significantly higher levels of aggression and vocalization [4]–[5] (own experience). Even extensively handled wild rats still demonstrate intense fear and aggression in response to human contact [6] (own experience). Their defensive behaviours are also different [7]. Furthermore, investigators report that laboratory rats demonstrate lower neophobia compared to their wild counterparts [8], [9], [10], [11] (own experience). Price [12] found that laboratory rats required less time to learn certain tasks, however their learned responses extinguished faster than in wild animals [13]. Wild rats are more sensitive to environmental changes early in life than their laboratory conspecifics [14]. Huck and Price [15] noted that wild rats are able to start climbing spontaneously even without prior experience, while laboratory rats require such experience for this type of activity.

Most of the data on this subject were collected a few decades ago, which is a sufficient reason to re-examine the issue of influence of laboratorization on the rat for at least two reasons. First, the continuing process of domestication/laboratorization could make the behavioural characteristics of laboratory rats even more distinct from their wild counterparts. Second, one can doubt that the wild population of *Rattus norvegicus* remains under the same pressure of the environmental factors, or alternatively, that nowadays environmental factors act in the same way as compared to the fifth or the sixth decade of XX century. Therefore, there is still a lot of scope for systematic comparative studies on the behaviour of these two groups of rats that may shed new light on the validity of current experimental procedures and tests.

One of the spontaneous and complex activities that could be observed in rats [16], [17], [18], [19] and many other rodents [18], [20] is burrowing. It is characterized by a high rate of individual differences and strongly affected by diseases, treatments, surgical interventions, etc. [17]. Studies on digging nesting burrows and underground tunnel systems are not very common, probably due to the limitations of laboratory settings, in which rats typically live in cages with floors that make digging impossible. Taking that into account, we should expect such a radical environmental change to affect rats bred in laboratories for multiple generations. The study of differences in this particular activity between laboratory and wild rats would thus seem a key component of a comparative strategy. Research on the subject was previously undertaken by Boice [16], who compared burrow systems of albino laboratory rats and wild Brown Norway rats in semi-natural and laboratory settings. He found no significant differences between the two strains in terms of size or configuration of their burrows. He also found no differences in the overall area and durability of burrow systems between animals raised in burrows and in laboratory cages. Boice noted that laboratory rats would rarely start digging without the presence of an object (such as a stone) to dig under, while wild rats were less dependent on this type of inducement. With limited research in this area, it seemed further analyses and experiments were needed.

A popular and well documented claim is that wild rats demonstrate significantly higher neophobia (avoidance of new objects in familiar surroundings [10]) than their laboratory counterparts [8], [9], [10], [11]. This is usually explained by the lack of predatory pressure and greater general stability of the laboratory setting. The absence of environmental pressures that presumably suppresses stimulus seeking in wild animals, may play a significant role in increased stimulus approach behaviour observed in laboratory animals. In addition, an intrinsically changeable habitat of wild rats may have generated different levels of avoidance towards specific categories of changes in that habitat. Placing a novel object in the experimental setting (e.g. a trap) may trigger neophobic behaviour in these animals, and consequently reveal the differences between strains.

Another interesting, and at the same time poorly researched aspect of rats’ behaviour is spontaneous swimming. In a laboratory setting, rats rarely come into contact with bodies of water giving them an opportunity to engage in this type of activity. Still, it is an established fact that rats are excellent swimmers as well as divers, and that they resort to swimming to get from point A to B and while foraging for food. They can swim, float, dive and swim under obstacles [21], [22], [23] (own experience). Rats’ ability to swim is used in various laboratory tests, primarily in memory, spatial learning and stress research, as well as in medical studies. The most popular setup is the Morris Water Maze [24], [25], [26], but other tanks are used occasionally [27], [28]. A common feature of all experiments of this kind is that they are conducted predominantly on laboratory rats. However, there are reasons to question the generalizability of their results for all rat populations [1], [29]–[30].

A related issue is the exclusive use of forced swimming in these experiments. This procedure may evoke intense stress in rats [31], and subsequently may significantly affect their physiological parameters [32], undermining the validity of research findings. Equally important is that subjecting animals to severe stress has profound consequences for their wellbeing and is in clear violation of the contemporary approach which advocates the use of low-stress procedures [33].

The purpose of the present study was to analyse species specific forms of behaviour (digging and swimming) and response to novelty in laboratory rats and their wild-type counterparts at a very early stage of laboratorization (1st-3rd generation). This is why we used rats from a laboratory colony created on the basis of wild population of *Rattus norvegicus* for the purposes of comparative studies - WWCPS [48]. The WWCPS strain was derived in 2006 from genetic material obtained from 5 independent free ranging colonies of feral rats.

Wistar rats for the study were obtained from Nencki Institute of Experimental Biology Polish Academy of Sciences and Medical University of Bialystok Centre for Experimental Medicine, and the Brown Norway and the Sprague-Dawley rats from Mossakowski Medical Research Centre of Polish Academy of Sciences. The WWCPS rats are bred by the present authors [34]–[36]. In our experiments, generations F1 to F3 of wild rats were used. This allowed for monitoring rats’ living conditions from their birth and eliminated the possible influence of stress caused by trapping as well as an adaptation process to the laboratory settings. Moreover, pairing individuals originating from various locations [36] resulted in obtaining WWCPS rats of low relatedness.

All procedures described in this paper were approved by the 1st and 4th Local Ethics Commissions in Animal Experimentation, Warsaw, Poland. All rats prior to experiments were housed in groups of 3 or 4 in Eurostandard type IV cages with ad libitum access to water and standard laboratory fodder.

## Experiment 1–Burrowing

### Materials and Methods

#### Subjects

The sample consisted of 12 experimentally naive rats (3 WWCPS males and 3 WWCPS females, and 3 Wistar males and 3 Wistar females) aged 3–4 months. The Wistar strain was chosen for comparison as one of the oldest and most popular laboratory rat strains. The experiment involved wild strain of rats (WWCPS) from F1 and F2 generations.

#### Equipment

**Figure 1:**
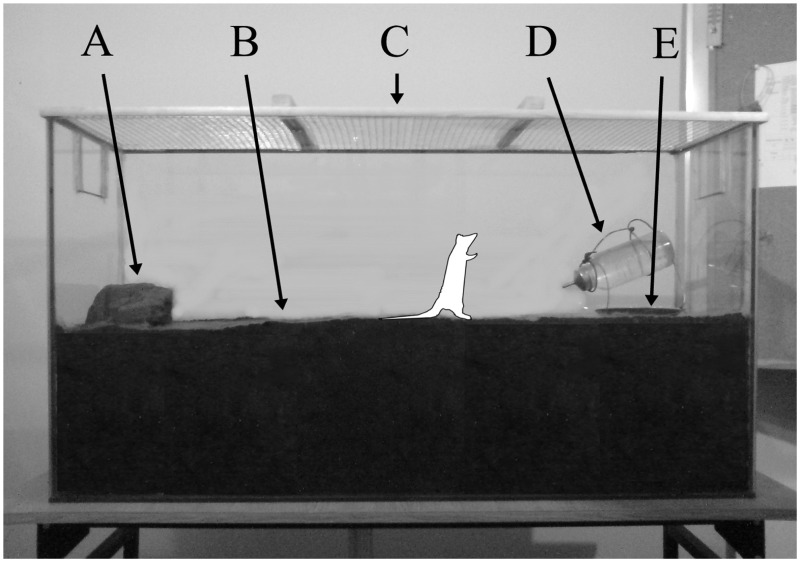
Tank used in the burrowing study. (A) digging stone; (B) soil mixture; (C) tank cover; (D) drinking bottle with wire stand; (e) feeding bowl.

A glass tank (Figure 1) 112 cm/60 cm/36 cm filled with a mixture of soils. The mix was a granulometric equivalent of sandy loam with a 10% humus admixture and was obtained from a field in the northern part of Warsaw district. Recording was made with 2 camcorders equipped with infrared illuminators connected to computers, allowing for simultaneous recording and real-time observation of tested animals from various angles.

#### Procedure

Immediately before being tested, the rats were moved from their maintenance room to the experimental room within their housing cages. The rats were placed individually in the experimental chambers for 6 days. Animals were provided with free access to a water bottle mounted on a wire rack and standard laboratory fodder in a metal bowl, which was refilled every second day of the study. To prevent the soil from drying, its top layer was dampened every second day with two litres of water from a garden sprinkling can. Each day the length and structure of burrows were measured from the outside (number of tunnel exits, number of underground chambers, number of forks in tunnels). The exact measurements were taken after removal an observed rat and the burrows had been excavated. The analysis also included behaviours such as moving nests to underground chambers and the stability of construction. The top layer of the soil was replaced at the end of each session (20% of total volume). The soil was completely replaced after every 3 sessions. Though there is a possibility of uncontrolled influence of remaining hormonal traces (left by previously tested animals) on the behaviour of subsequent subjects, we have taken steps to minimize the role of this possible effect. The soil used for the purpose of the study was mixed at the beginning of the experiment with the soil used in the pilot sessions (except the top layer, which was always initially clean), which standardized experimental conditions for all animals. This part of the experimental procedure closely resembled an experiment conducted by Boice [16], as it was our main goal to replicate his study.

The rats’ behaviour was recorded continuously with camcorders and software featuring zone motion detection. The rats had constant access to water and food. The tank contained a digging stone measuring 15 cm/20 cm/5 cm. On the second day of testing, the rats were provided with nesting material. The experimental chamber was kept at a constant temperature (20°C) and humidity (50%). The day/night cycle was set to 12 h/12 h.

### Results

**Figure 2:**
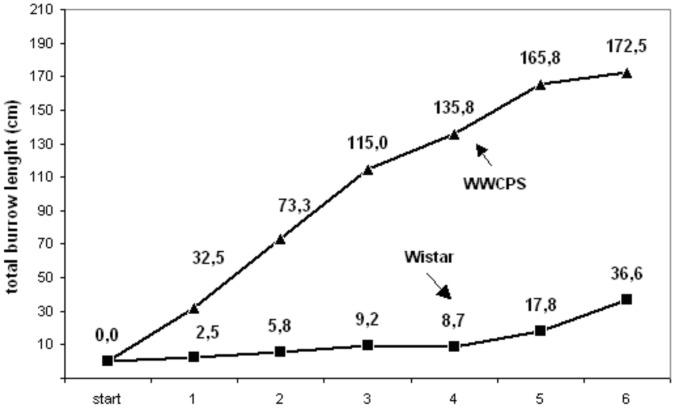
Mean length of burrows dug by rats from either strain for each testing day.

Wild rats are faster at building tunnels (length in cm/day) than Wistar rats. Due to the sample size (N = 12) analysis was done using non-parametric Mann-Whitney U test. Differences were significant for each testing day, see figure 2.

**Figure 3:**
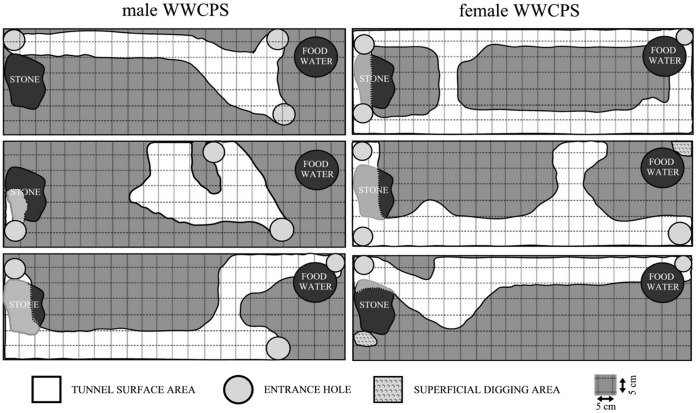
Diagram showing tunnels and burrows dug by wild WWCPS rats (overhead view).

**Figure 4:**
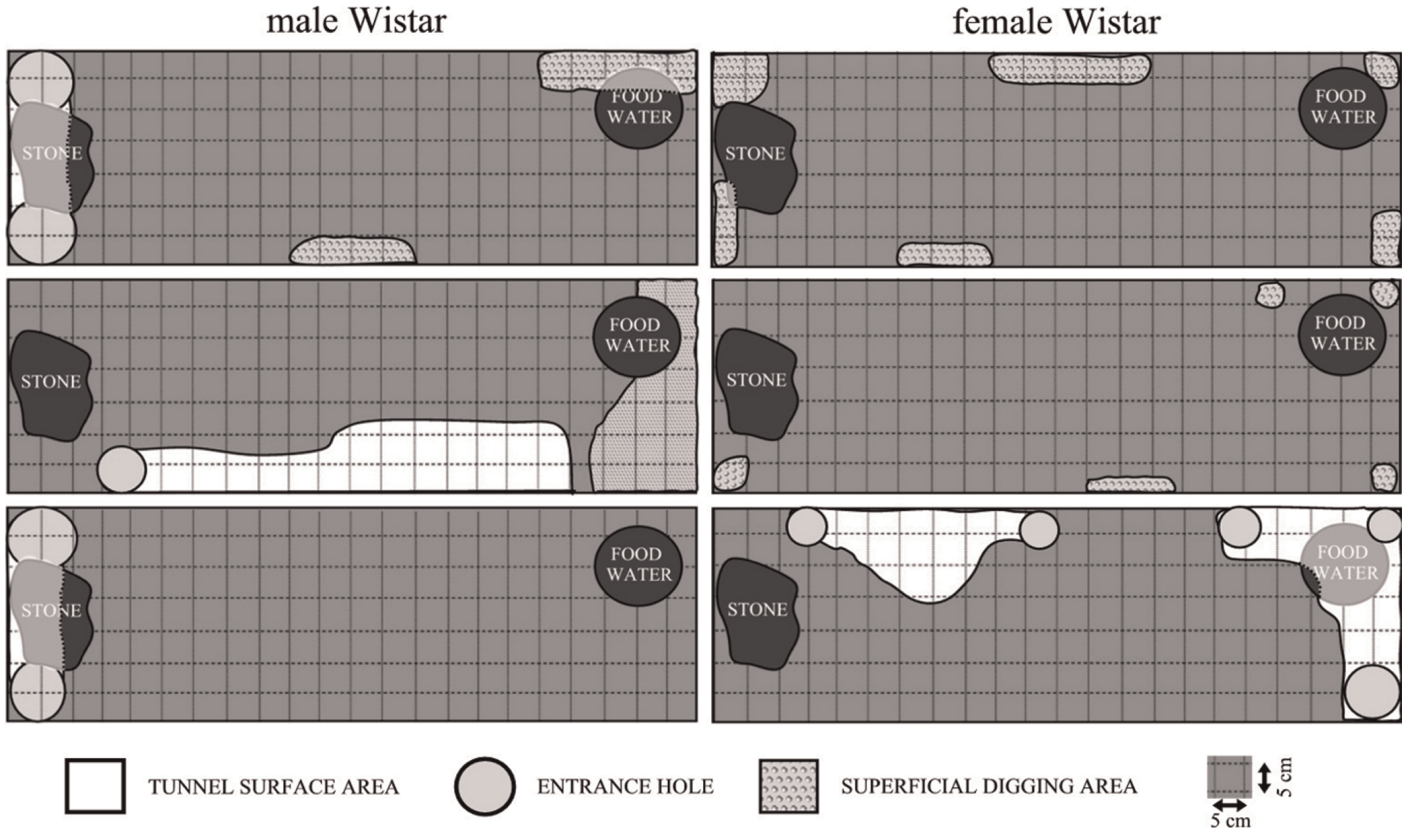
Diagram showing tunnels and burrows dug by Wistar rats (overhead view).

Wild rats construct more complex burrow systems than Wistar rats (Figure 3 and Figure 4). The measure of burrow complexity was the number of chambers, the number of forks in tunnels (the number of points with at least 3 tunnels connecting) and the number of exits (measured at the conclusion of the test). Only 2 laboratory rats dug tunnels that afforded them full cover for the whole body. We identified at least one fork in the burrows constructed by all wild rats (M = 1.17). The mean number of chambers in wild rats’ burrows was M = 1.66 (SD = 0.82, min = 1, max = 3). The tunnels dug by wild rats, in contrast to those constructed by laboratory rats, invariably formed one interconnected structure and always featured at least 2 exits. No qualitative differences between strains were found in the method of digging. All rats in the studies widened their tunnels by breaking up the soil with their teeth followed by pushing it back with their front and rear paws, throwing it outside from time to time and creating a pile close to the burrow’s exit.

All wild (WWCPS) rats in the experiment dug burrows and moved their nests to them (see video S1 and video S2 to obtain a general view of this behaviour). Out of two laboratory rats that constructed tunnels, only one female made her permanent residence underground. Wild rats were more likely (at the level of statistical trend) to live in their burrows than their laboratory-raised conspecifics (U = 3, p = 0.083).

There were no differences in terms of tunnel length between males and females (U = 18, p = 1.00), both in wild (U = 3.5, p = 0.658) and laboratory rats (U = 3, p = 0.513).

Tunnel collapse was observed in two out of four laboratory rats that started digging. These rats dug under the object placed in the experimental tank, and its weight caused the niches constructed by the animals to collapse. No such events were observed in the six wild rats in the study.

## Experiment 2–Neophobia

### Materials and Methods

#### Subjects

The sample consisted of 12 rats (3 WWCPS males and 3 WWCPS females, and 3 Wistar males and 3 Wistar females) aged 3–4 months, which served as subjects in Experiment 1.

#### Equipment

A 112 cm/60 cm/36 cm glass tank (Figure 1) filled with a mixture of soils. The mix was a granulometric equivalent of sandy loam with 10% of humus admixture (see: Experiment1–Burrowing). Live capture traps consisting of a small metal cage with a platform triggering the trap mechanism inside were placed in the tank. The platforms were baited. Recording was made with 2 camcorders equipped with infrared illuminators connected to computers, allowing for simultaneous recording and real-time observation of the animals in the experiments from various angles.

#### Procedure

Seven days after the start of the burrowing experiment (see: Experiment 1–Burrowing), a live capture trap was placed in the tank. It was baited with standard fodder (Labofeed H), paper towels, and cheese. The study endpoint was either the capture of the rat or the lapse of 7 days.

During the experiment, the number of animals caught in traps and time to capture were recorded.

### Results

On the basis of chi-square values we can conclude that Wistar rats were trapped more often than WWCPS rats χ^2^(1, N = 12) = 6 (Table 1).

**Table 1:** Number of captured rats and chi-square values in selected time intervals.

	within7 min	within1 hour	within4 days	within7 days
Wistar rats	6 (100%)	6 (100%)	6 (100%)	No change
WWCPS rats	0 (0%)	1 (17%)	2 (33%)	No change
χ^2^	12.00	8.57	6.00	No change
df	1	1	1	No change
p	0.001	0.003	0.014	No change

Laboratory rats (M = 4.5 min.) were caught in a significantly shorter period of time than the 2 captured wild rats (M = 2900 min): U = 0, p = 0.046.

Laboratory rats explored by almost immediately entering the cage, which resulted in their immediate capture. Wild rats took more time and were more careful in exploring the trap. Initially, they explored the traps from the outside, which is why they set them off without walking into them more times than laboratory rats. Observation showed that most wild rats, when exploring the inside of the trap, kept low to the ground (stretch attend posture) in a position allowing immediate retreat.

## Experiment 3–Swimming

### Materials and Methods

#### Subjects

On account of differences between wild and laboratory rats that were observed in the above experiments, we decided to engage other laboratory strains in the next study. We assumed that it would provide us with further data concerning between-strains differences and allow us to generalize the results on the laboratory rat population. We decided to add another albino strain (*Sprague-Dawley*) (popular experimental subject, however, characterized by the properties rarely found in nature – see for example: [37], [38], [39] and a pigmented strain (*Brown Norway*) as more similar morphologically to wild counterparts.

The sample consisted of 72 rats aged 4.5–9 months. They were divided into four groups based on strains. The first group (Wild) consisted of wild rats of the WWCPS strain (12 males and 12 females). The second group (BN) consisted of Brown Norway rats (6 males and 6 females). The third group (Wistar) were laboratory rats of the Wistar strain (12 males and 12 females). The fourth group (SD) were laboratory rats of the Sprague-Dawley strain (6 males and 6 females). The experiment used wild WWCPS strain rats from F1, F2 and F3 generations. None of the rats in the study had previous contact with an open body of water or an opportunity to swim. Prior to the study, all rats were group-housed with constant access to food and water. The day/night cycle was 12 h/12 h.

**Figure 5:**
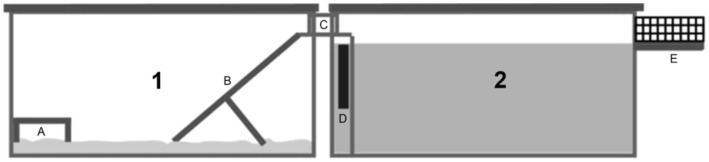
Experimental apparatus. 1– housing tank; 2– water-filled tank (A – shelter, B – plank, C – passage between tanks, D – heater and filter, E – cage).

#### Equipment

The experiment used two identical, connected glass tanks (Figure 5), measuring 112 cm/60 cm/36 cm each. One glass tank was used to house the rats in the study. A wooden box was placed inside that tank to provide shelter and a wooden plank at approximately 45° angle was installed to allow access to the passage connecting the two tanks. The second tank, which was the experimental chamber, was filled with 48 cm of water. The rats had constant access to the tank filled with water through the passage connecting the two tanks. A small ladder was placed at the edge of the water. The water-filled tank had a small cage attached opposite the entrance from the other tank. Both tanks were covered with grating to prevent the animals from getting out. Recording was made with two camcorders equipped with infrared illuminators connected to a computer, allowing for simultaneous recording and real-time observation of the animals in the experiments from various angles.

**Figure 6:**
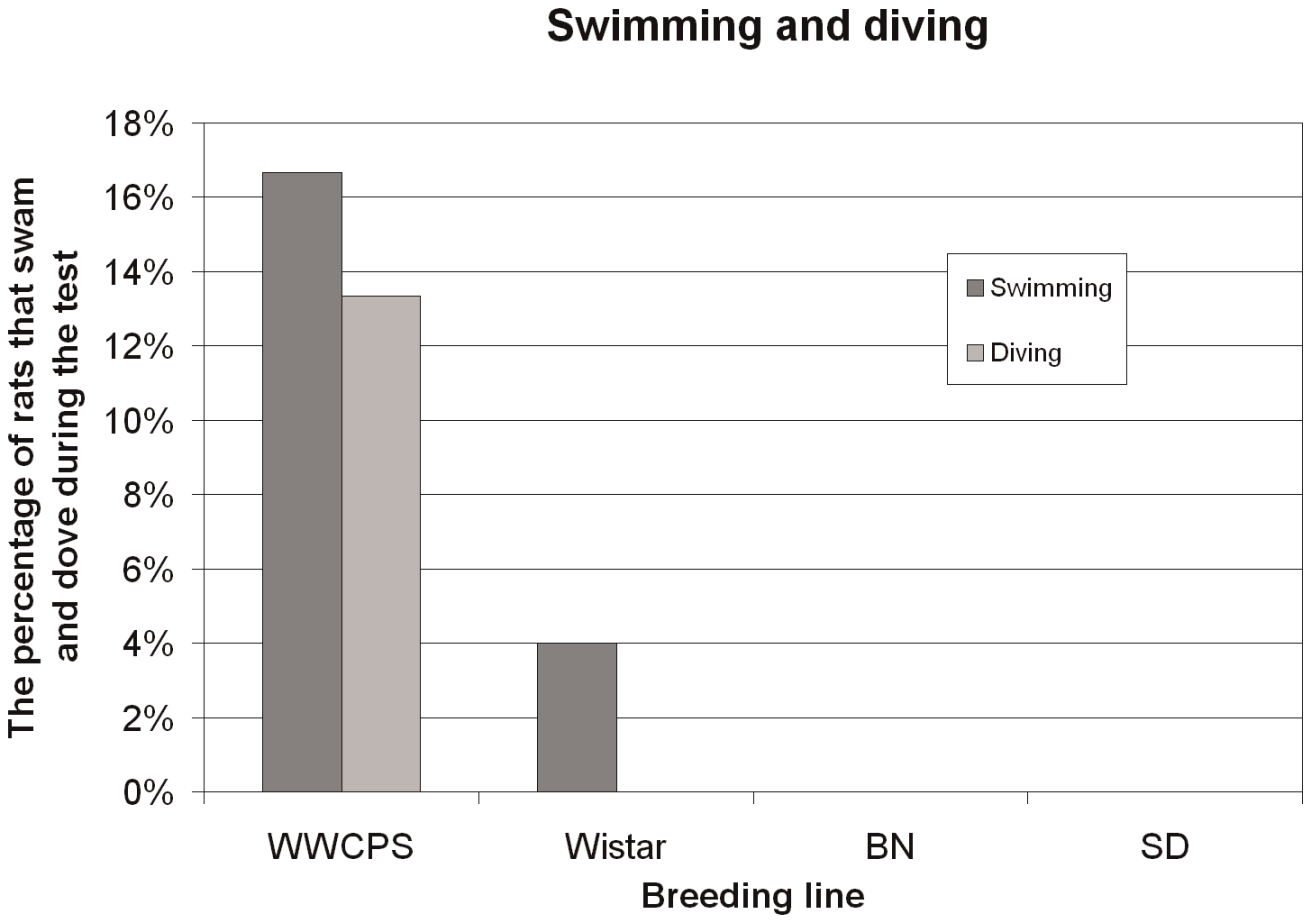
The percentage of rats that swam and dove during the test.

#### Procedure

The temperature in the experimental room was 22°C. Water temperature during the experiment was 21°C. Immediately before being tested, the rats were moved from their habitation room to the experimental room within their housing cages. Three rats of the same sex from one strain were placed in the housing tank, selected randomly from the rats that shared the housing cage prior to the experiment. The animals were left undisturbed in the tanks for the period of 72 h. Throughout the stay in the experimental area, the rats had unlimited access to the water-filled tank and constant access to food and water (present in the water tank only). The daily cycle in the experimental room was set at 12 h/12 h. Camcorders were used to record the rats’ activity in the water-filled tank.

The following variables were measured during the experiment: time to dipping the head under water; time to swimming; number of times the tank was crossed; time to diving; number and time of dives; other activity within the water-filled tank, i.e. the number of behaviours involving sniffing above the water and near the objects in close proximity to water (partition, plank, etc.) and dipping front paws in the water. The above measurements were done by way of analysing video material recorded during the experiment.

### Results

During the study, most of the laboratory rats made no attempts to cross the water, only approaching its surface. The behaviour within the water-filled tank was limited to drinking, dipping front paws in the water and sniffing above water surface and near objects located in close proximity of the water. Whereas wild rats demonstrated a wide array of swimming behaviours (see video S3 to obtain a general view of this behaviour). Significant differences between laboratory strains were also found.

#### Swimming

**Figure 7:**
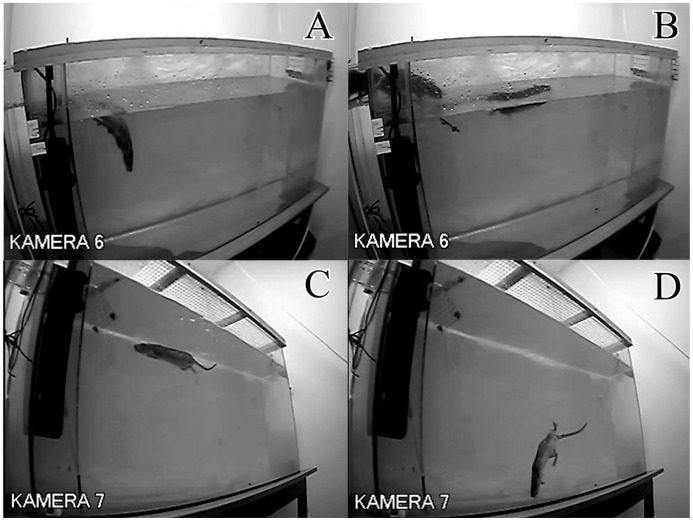
Female wild rat (WWCPS). (A) submerging a significant portion of her body in water; (B) swimming across the water-filled tank; (C) swimming horizontally underwater; (D) diving.

Spontaneous swimming was observed in three groups of wild rats and only in one group of laboratory rats (single Wistar female) – figure 6. After initial probing by dipping front paws and the front part of the body with head kept above water, rats left the starting platform (figure 7B) and swam across the water (approximately 60 cm), and then entered the cage placed at the other side of the tank, outside of the water area. There was noticeable variation in latency of swimming among individuals. One of the wild rats entered the water immediately after onset of the experiment, while other rats started swimming after several hours of probing behaviours e.g. dipping front paws in the water.

#### Head dipping

Another behaviour observed in wild rats was repeatedly dipping the head in the water, often combined with submerging a large portion of the body to a considerable depth, but never losing contact with the ground (figure 7A). This behaviour usually did not lead to swimming or diving. Behaviour of this type was not observed in laboratory rats.

#### Diving

Only wild rats spontaneously dove into the water during the experiment (figure 7C and 7D) – figure 6. While swimming, the animals reached the bottom of the tank multiple times and several times changed direction under the surface of the water. None of laboratory animals demonstrated such behaviour (figure 7).

#### Activity within the water-filled tank

**Figure 8:**
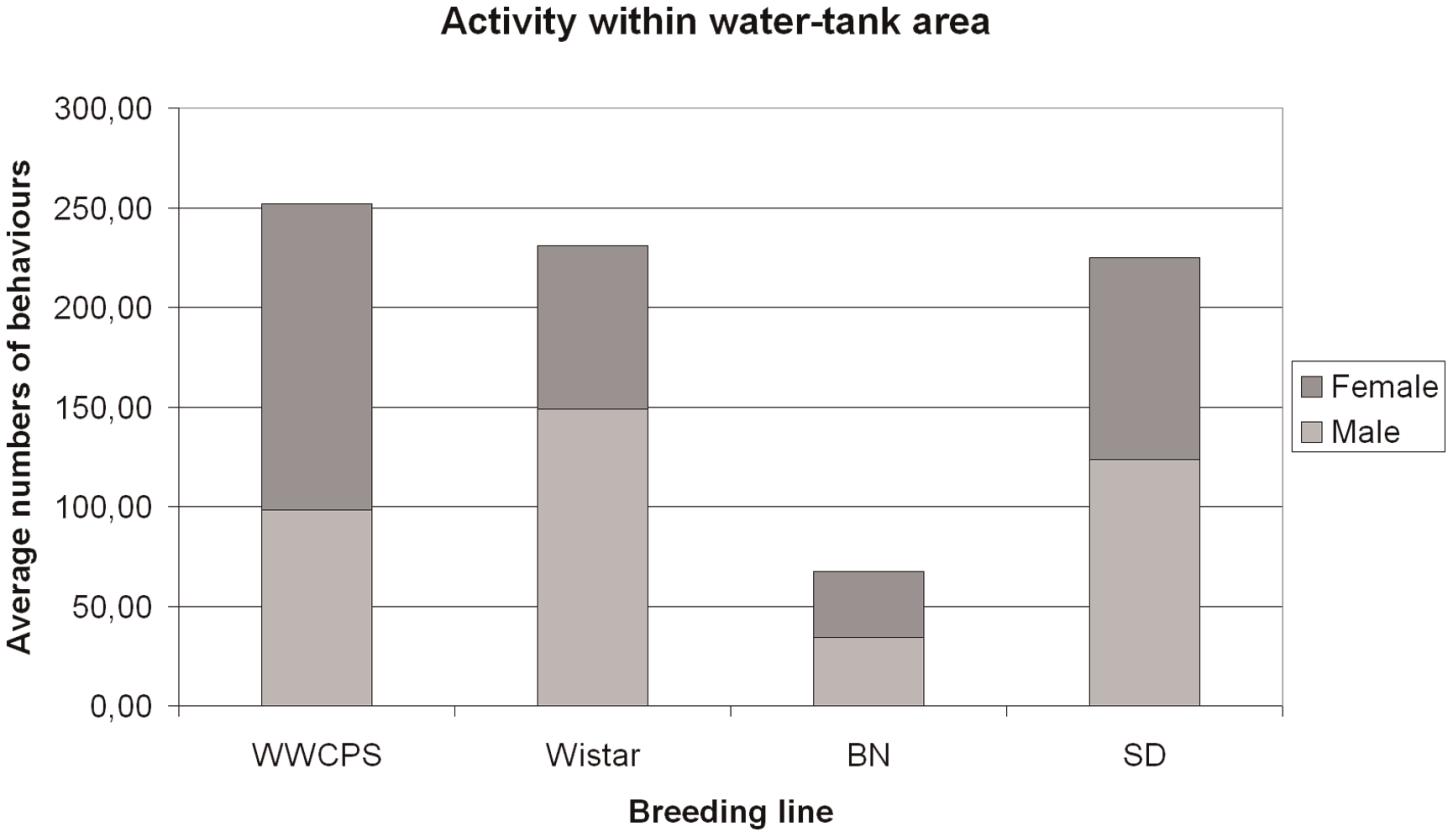
The rats’ activity within the water-filled tank.

The measure of the rats’ activity within the water-filled tank was the number of actions involving sniffing above the water and near the objects located in close proximity of the water (partition, plank, etc.). These behaviours were observed in all groups of rats in the study (figure 8).

Strains differed significantly in terms of this type of activity (ANOVA *F*(3;68) = 11.832, *p*<0.001). However, *post hoc* analysis using Tukey’s test showed that the significance of that difference is accounted for by the difference between the BN strain and the other strains. There were no significant differences in activity between Wild, Wistar and SD strains (*p*>0.05).

Similar results were obtained for dipping front paws in the water. There were also no significant differences in the quantity of that behaviour between Wild, Wistar and SD strains (*p*>0.05), whereas BN rats did not demonstrate such behaviour at all.

No significant differences between sexes in activity within the water-filled tank or dipping front paws in the water were observed.

#### Other activity within the water-filled tank

Wild rats (both males and females) crossed the length of the water-filled tank by climbing the grated cover to reach the cage at the opposite end of the tank. During those crossings, they fell in the water several times and immediately escaped from the water. Only one group of the laboratory rats (single Wistar female) demonstrated this type of behaviour.

## Discussion

In light of earlier research e.g.: [1], [4], [7], [9], [40], [41], [42], [43], [44], [45], [46] it seems evident that domestication changes animal morphology and behaviour.

Domestication of rats by adapting them to laboratory conditions resulted in numerous scientifically proven behavioural changes that may have significant effects on experimental findings, and their extrapolation to the general population [1], [29], [47].

Our experiments were designed to test the effects of domestication on rarely investigated aspects of rats’ behaviour, such as digging and swimming, as well as neophobia levels. We insisted on using low-stress procedures and spontaneous behaviour.

The experiment on burrowing, despite a small sample, showed significant differences with respect to this activity. The tunnels dug by wild rats were over 5 times longer than those built by laboratory rats. Within the first few hours into the experiment all wild rats constructed a safe underground nest, which, in a natural setting, would offer comprehensive protection in the event of an attack by a predator. Only two (one-third) of Wistar rats constructed burrows in which they could live; these, however, were much smaller, and only one female made her permanent dwelling underground after as many as 5 days of testing. It should be noted that out of the four Wistar rats that constructed any burrows at all, two dug under the objects placed in the tank in a way that made them collapse into the tunnel and onto the digging rat.

Our findings contradict the results obtained by Boice [16], who found no differences in digging behaviour between the strains in his study. In the course of his research, he concluded that the tunnelling ability in rats is species specific, and thus present across all strains and independent of the environment in which they had been raised for generations. However, taking into account the fact that those studies were conducted several decades ago, we should consider the possible effect of altered environmental conditions and selective breeding of laboratory rats in that period on their behaviour. Our results suggest that the burrowing and tunnel-digging pattern is partially extinct in at least one of popular laboratory rat strains and general conclusions about the whole species, including wild rats, on the basis of their behaviour may be unfounded.

There were also major differences found in the neophobia experiment. Placing a novel object (in this case, a trap) in a familiar environment revealed both quantitative and qualitative behavioural differences. Wild rats demonstrated a much wider array of exploratory behaviours, and much more cautious approach to the potentially dangerous object. This resulted in very few cases of wild rats being caught in the trap. Such a high level of neophobia in wild rats is consistent with previous data [8], [9], [10], [11]. The specifics of a laboratory setting with its stability and lack of outside threats may have diminished the level of neophobia in rats bred in laboratories for a number of generations. To discover the mechanism of that change is a particularly interesting task. It seems that studies on gradual extinction of neophobia in domesticated animals may shed new light on the mechanisms of evolutionary change on the one hand, and epigenetic processes on the other.

Some profound differences between wild and laboratory rats also emerged in the swimming experiments. The activity of most of laboratory rats in the water-filled tank was limited to drinking, sniffing and dipping front paws in the water. Generally they made no attempts to get across the water (except a single Wistar female), whereas wild rats showed a wide range of spontaneous behaviours related to swimming. Not only did they enter the water, but they also deeply plunged their heads, swam and floated on the water, as well as dove reaching the bottom of the tank. Moreover, both female and male WWCPS rats tried to cross the water without entering it, by climbing on the protective grating. Furthermore, significant differences with respect to the activity within the water-filled tank were seen primarily between three laboratory strains in the study. It was the Brown Norway rats that demonstrated the lowest activity (sniffing, dipping front paws in the water), while this behaviour of Wistar rats and Sprague-Dawley rats was more similar to this behaviour in their wild counterparts.

It seems to be reasonable that one could explain behavioural differences in burrowing, getting caught in traps, and swimming by reference to fear reduction. Laboratory rats live in a low-stress and stable environment. Perhaps the higher level of stress in wild rats motivates them to dig and swim in search of shelter and/or to discover ways of escaping. In the wild, once they leave the nest, they are subjected to predation, which lead them to flight to any place of concealment [48]. Although wild rats actively explore the surroundings, their activity is often suppressed/modified by increased levels of anxiety [48]. Furthermore, laboratory animals, used to constant availability of food and never having been directly confronted with a predator or intruder, may lack some behaviours essential for survival. Bred for hundreds of generations in an environment that does not require moving around, they may be less motivated to engage in complex and energy-consuming behaviours, such as digging and swimming. After several decades of breeding in laboratory conditions, a drastic departure from any natural setting, the rats’ behavioural repertoire has been diminished with various behaviours being completely eradicated [45]. A hypothesis of an epigenetic nature of these changes seems to be relevant [49]. Consequently the research on differences in the behaviour of animals with varying degrees of domestication, may prove to be important for understanding the plasticity of behaviour in general terms.

One may argue, that the concept of rat’s line/strain characteristic is speculative and misleading. There is a reasonable possibility, that various factors, that are specific for given region, may alter (through selection or/and epigenesis) behaviour of *Rattus norvegicus* inhabiting different places of the world. It is also possible, that specific standards of laboratory facilities, varying across countries and continents, may play their role in this respect.

While burrowing appears to be an activity that involves an optional utility in the sense of preparing nests and a system of tunnels to facilitate escape, swimming may be an emergency strategy, allowing rats to escape immediately by crossing a body of water in the event of danger, or an acquired strategy of finding food [21], [22], [23]. The lack of regularity in spontaneous swimming behaviour, as well as its relatively low rate may be associated with an insufficient level of danger encountered by the animals in the experiment. Staying in close proximity of a water-filled tank, constant access to it, relatively long time of the experiment and lack of immediate danger may have reduced the level of fear in the animals to the point where most of them did not need to use this route of escaping. The conclusion could be that rats, despite being excellent swimmers, rarely engage in this activity spontaneously, unless forced to in order to escape or find food. Even though the study of spontaneous behaviour appears to afford more reliable data for generalizing onto populations living in natural conditions and helps protect the animals’ well-being, it presents considerable challenges when it comes to behaviour motivated by fear.

The differences between laboratory strains in terms of activity within the water-filled tank observed in the experiment suggest that the choice of particular study subjects from among domesticated animals may influence the results of research. Various strains of laboratory rats may differ in some aspects from one another to a greater extent than they differ from their wild conspecifics.

## Supporting Information

Video S1 Male wild-type rat’s initial digging.
(MPG)

Video S2 Female wild-type rat digging inside a burrow.
(MPG)

Video S3 Female wild-type rat diving in a water tank.
(MPG)
